# The stability of phenolic compounds and the colour of lingonberry juice with the addition of different sweeteners during thermal treatment and storage

**DOI:** 10.1016/j.heliyon.2023.e15959

**Published:** 2023-05-03

**Authors:** Kjersti Aaby, Mathias Rudolf Amundsen

**Affiliations:** a Nofima AS, Norwegian Institute of Food, Fisheries and Aquaculture Research, Osloveien 1, N-1431, Ås, Norway; b Department of Arctic and Marine Biology, UiT The Arctic University of Norway, NO-9037, Tromsø, Norway

**Keywords:** *Vaccinium vitis-idaea* L, Anthocyanins, Flavonols, Flavan-3-ols, Hydroxycinnamic acids, Sucrose

## Abstract

Lingonberries (*Vaccinium vitis-idaea* L.) are rich in phenolic compounds associated with several health benefits. The berries are also astringent, sour, and bitter and the addition of a sweetener is necessary to increase the palatability of lingonberry products. The addition of a sweetener may, however, affect the stability of phenolic compounds in the product. The aim of this study was thus to determine the effects of the addition of sweeteners (sucrose, acesulfame K or sucralose) and temperature on the stability of anthocyanins, flavonols, flavan-3-ols, hydroxycinnamic acids and the colour of lingonberry juice during thermal treatment and storage. The addition of sweeteners did not affect the stability of phenolic compounds or the colour of lingonberry juice during thermal treatment or storage. The stability of the phenolic compounds was significantly affected by temperature. Anthocyanins were the least stable of the phenolic compounds. The half-lives of total anthocyanins were 3.8, 2.0 and 0.8 h at 75, 85 and 95 °C, respectively. The half-lives during storage were 12.8 and 2.7 weeks at 6 and 22 °C, respectively. Cyanidin-3-galactoside, the major anthocyanin in lingonberries, was extensively degraded during storage, probably due to galactoside side-activities of the enzyme preparation used in juice production. After thermal treatment, the juices were darker and bluer, with lower chromaticity, while after storage, the juices were lighter, more yellow, and had higher chromaticity.

## 1 Introduction

Lingonberries (*Vaccinium vitis-idaea* L.) are among the most important non-wood forest products in the Nordic countries [1]. Traditionally, lingonberries have been produced into jams, which are considered a delicacy in Nordic cuisine. Lingonberries contain high concentrations of phenolic compounds, which are associated with several health benefits [[2], [3], [4]], and some studies have suggested an inverse association between high flavonoid intake (usually higher than 500 mg per day) and occurrence of cardiovascular diseases [3]. The phenolic compounds found in lingonberries are anthocyanins, flavonols, flavan-3-ols, and hydroxycinnamic acid derivatives (HCAs) [[5], [6], [7]]. Of these compounds, the anthocyanins are of especial interest both because they provide the scarlet red colour of the berries and because they are associated with several positive health benefits [8,9]. Consequently, the anthocyanins have been studied in many matrices including e.g. pomegranate [10] and blood orange juice [11], in addition to in several berry species [[12], [13], [14], [15], [16], [17], [18]].

Lingonberries are seldom consumed as fresh berries but are produced into products such as jams. Cranberry (*V. macrocarpon* Ait.) juice, which has many of the same properties as lingonberry juice, is common in supermarkets around the world, and it is likely that lingonberry juice could also be a successful product. However, more knowledge about the chemical constituents in lingonberry juice and the stability of health-related compounds during processing and storage is needed. Several food processing steps involve high temperatures to increase yield, extend shelf life or create products with desired properties. In juice processing, the berries are exposed to elevated temperatures during enzymatic mash treatment, typically 45–50 °C for 1–2 h, and the juice is pasteurized, where temperatures up to 95 °C are normally applied [19]. Exposure to high temperatures for longer periods is shown to be detrimental for phenolic compounds, with anthocyanins being the most labile [[20], [21], [22]]. The degradation rate of anthocyanins increases with increasing temperature, although the degradation rates are highly dependent on the source of anthocyanins and the matrix [16,23]. Factors that affect the stability of anthocyanins, in addition to temperature, are pH, ascorbic acid content, degrading enzymes and availability of oxygen and light during processing and storage [24]. Anthocyanins are more stable at low pH, where anthocyanins mainly occur in the flavylium cation form. Ascorbic acid is found to destabilize anthocyanins and the colours of berry juices and syrups [16,25]. Although phenolic compounds are degraded during juice processing, the most severe degradation is often found during the storage of berry products [26]. Temperature and duration were the main factors affecting the degradation of anthocyanins in berry juices during storage in prior studies [13,14,18,20]. The degradation rates of anthocyanins in juices made of various berries differed, illustrating that the anthocyanins stability is affected both by their structure and the berry species where they are present. Furthermore, the stability of anthocyanins is dependent on other ingredients and processing conditions, as illustrated by mixtures of blackcurrant juice and other juices [13].

Due to the instability of anthocyanins, many studies have been conducted to find means to increase the stability of these compounds. Encapsulation and association reactions, such as co-pigmentation, are methods used to stabilize anthocyanins [24,27]. The addition of hydroxycinnamic acids to berry juices stabilized both anthocyanins and the colour of the juices during storage [28]. The addition of other ingredients, including sugars, may also influence the stability of anthocyanins and other phenolic compounds [16,[29], [30], [31], [32]]. Sucrose is the most utilized sweetener in berry products, but other sweeteners are also used. Among them are high-intensity sweeteners, such as sucralose and acesulfame-K, which do not add calories to the product. The effect of sweeteners on anthocyanin stability is variable and depends on the type of sweetener, concentration of sweetener, matrix and temperature used [16,[29], [30], [31], [32], [33], [34]]. Fructose is shown to destabilize anthocyanins in berry juices both during thermal treatment and storage, while sucrose increased stability, decreased stability, or had no effect on anthocyanin stability [29,30,33,34]. Acesulfame K stabilized anthocyanins in cornelian cherry juice at 75 °C but not at the lower temperatures investigated [30].

Anthocyanins are responsible for the red colour of berry products. As anthocyanins degrade during heat treatment and storage, it is expected that the colour of the product will change. It was previously shown that the colour of berry juices became more yellowish and lighter and had lower colour saturation during storage, but with varying colour development in the different juices [28]. The colours of jams of strawberries and raspberries were affected differently by thermal processing and storage [35]. While a darker colour was observed in both jams after processing, the strawberry jam developed a slightly lighter and more yellow colour during storage, and the raspberry jam became more bluish. The colour stability of anthocyanin-rich elderberry concentrates increased with the addition of sucrose during storage, while minor effects were observed for the colour stability of black currant concentrates [36].

Although the degradation of phenolic compounds, especially anthocyanins, has been extensively studied, the effect of temperature during thermal processing and storage of lingonberry juice supplemented with different sweeteners has not been investigated. As lingonberries are perceived as bitter, acidic, and astringent [37], the addition of a sweetener to increase the palatability of lingonberry products is necessary. If a sweetener could additionally increase the stability of phenolic compounds and colour, it would be advantageous. As studies on the effect of sweeteners on the stability of anthocyanins diverge depending on anthocyanin composition and matrix, among other factors, it is essential to study the effects in the actual product. It is important to understand how different treatments and the addition of ingredients affect phenolic compounds, especially anthocyanins, to preserve these compounds and extend the sensorial quality and health benefits of the product. Thus, the aim of this study was to determine the effects of sweeteners (sucrose, acesulfame K or sucralose) and temperature on the stability of anthocyanins, flavonols, flavan-3-ols, hydroxycinnamic acids and the colour of lingonberry juice during thermal treatment (50, 75, 85 and 95 °C for 2 h) and storage (6 and 22 °C for 16 weeks). The working hypothesis was that the sweetener would affect the stability of phenolic compounds in lingonberry juice during thermal treatment and storage.

## 2 Materials and methods

### 2.1 Chemicals

A pectinase enzyme preparation (Rohapect MC, AB Enzymes GmbH, Darmstadt, Germany) was used for juice production. Sucralose (E955) and acesulfame-K (E950) were obtained from Haarla Oy (Tampere, Finland). The standards analysed were cyanidin-3-galactoside and cyanidin-3-glucoside (Polyphenols AS, Sandnes, Norway); 3,4-dihydroxybenzoic acid (protocatechuic acid) (Fluka, St. Gallen, Switzerland); procyanidin A2 dimer and procyanidin B2 dimer (Extrasynthese, Genay, France); and 3-caffeoylquinic acid (chlorogenic acid), quercetin, 2,4,6-trihydroxybenzaldehyde (phloroglucinaldehyde), *p*-coumaric acid, quercetin-3-rhamnosyl glucoside (rutin), (+)-catechin and (−)-epicatechin (Sigma‒Aldrich, Missouri, USA). All solvents used were of HPLC-gradient grade, and water was of Milli-Q quality (Millipore Corp., Cork, Ireland).

### 2.2 Berry material and juice processing

Lingonberries (*V. vitis-idaea* L.) were picked in the wild in southern Norway (60°3 N, 12°0 E) the autumn 2019 and provided by Norwegianberries AS (Birkenes, Norway). The berries were stored at −20 °C until processing in August 2020. To inactivate endogenous enzymes in the berries, frozen lingonberries (31 kg) were heated in a steam oven (Electrolux-air-o-steam, Electrolux Professional GmbH, Tübingen, Germany) until the temperature reached 80 °C in the core of the berries and was held for 2 min. The berries were cooled to 50 °C, and the pectinase enzyme preparation (0.165 g per kg of berries) was added. The enzymatic treatment was performed with constant stirring (Classic Proline Touch-Mix 100 L, Classic Gastro A/S, Fredericia, Denmark) at 50 °C for 1 h and 20 min. Pressing of the berry mash was performed through a fine-meshed cloth in a packing press (50 P1, Voran, Pichl bei Wels, Austria) at 150 bars. The juice yield was 73%.

The juice was diluted with water (3/1, w/w) to make a juice stock for the stability experiments.

For the heat stability experiment, diluted juice was divided into two groups: one with 10% sucrose (SUK) and one unsweetened juice (US). The juices were poured into polypropylene tubes (15 mL) with high density polyethylene caps (Sarstedt AG & Co. KG, Nümbrecht, Germany) and heated in water baths at 50, 75, 85 and 95 °C for 2 h. Sampling in triplicate was performed after 0, 5, 15, 30, 60 and 120 min followed by rapid cooling in ice water.

For the storage stability experiment, potassium sorbate (0.4 g per kg) was added to the diluted juice. The juice was then divided into four groups, that is, one unsweetened juice (US) and three juices with added sweeteners mimicking the sweetness of 10% sucrose: sucrose 10% (SUK), sucralose 0.017% (SUC) and acesulfame K 0.057% (ACE). The concentration of added sucrose (10%) were based on the results of a preliminary sensory test with 5, 10 and 15 g sucrose per 100 g juice and previous studies on ideal sweetness of juices [38]. The concentrations of the high-intensity sweeteners was based on their sweetness potency previous determined, to mimic the sweetness of 10% added sucrose [39]. The juice was stored in polypropylene tubes (15 mL) at 6 °C and 22 °C in the dark. Sampling of the juices was performed in triplicate after 0, 2, 6 and 16 weeks.

The heat and storage stability experiments were performed once for each temperature. Comparable experimental designs have been used in testing of stability in juices of other berries [13,14]. However, for more robustness of the results, the experiments should have been repeated with more than one batch of lingonberry juice.

After sampling in both experiments, the tubes were centrifuged at 4654 *g* for 10 min at 20 °C (Heraus Multifuge 4 KR, Kendro Laboratory Products GmbH, Hanau, Germany), and the supernatants were collected. Samples for HPLC analysis were filtered through Millex HA 0.45 μm filters (Merck Millipore Ltd., Cork, Ireland) and stored in glass vials at −80 °C until analysis.

The concentrations of chemical constituents in the SUK juice were 90% of the concentrations in the other juices on a weight basis (per 100 g). Due to the increased density (from 1.045 to 1.075 g/mL) with the addition of 10% sucrose (w/w), the concentrations of chemical constituents in the SUK juice should be ca. 93% of the other juices on a volume basis (per 100 mL).

### 2.3 Soluble solids, pH, and titratable acidity

The supernatants of the juices were analysed for soluble solids (SS), pH and titratable acidity (TA). SS was determined using a digital refractometer (RE40, Mettler Toledo, Greifensee, Switzerland) and expressed as °Brix (%). pH was determined with a pH metre (827 pH lab., Metrohm, Herisau, Switzerland). TA was measured by titrating the supernatant (4 g) diluted with distilled water (total weight 40 g) with 0.1 M NaOH to pH 8.0 using an automatic titrator (T50 Titrator, Mettler Toledo). The concentration of TA was expressed as g citric acid equivalents per 100 g. The samples were analysed in duplicate.

### 2.4 Analysis of phenolic compounds by HPLC-DAD-ESI-MS

The analysis was performed on an Agilent 1100 series HPLC system (Agilent Technologies, Waldbronn, Germany) equipped with an autosampler cooled to 4 °C, a diode array detector (DAD), and an MSD XCT ion trap mass spectrometer fitted with an electrospray ionization (ESI) interface. The samples from one experiment, that is, the heat or the storage stability experiment, were analysed in one batch in randomized order. The phenolic compounds were separated at 40 °C on a Synergi 4 μm MAX RP C12 column (250 mm length × 2.0 mm i.d., 4 μm particle size) equipped with a 5 μm C12 guard column (4.0 mm length × 2.0 mm i.d., 5 μm particle size), both from Phenomenex (Torrance, California, USA). The mobile phases used were A; formic acid/water (2/98, v/v) and B; acetonitrile in the following gradient: 0–10 min 5–10% B, 10–22 min 10–12.4% B, 22–42 min 12.4–28% B, 42–50 min 28–60% B, 50–55 min 60% B, and 55–58 min 60-5% B with a solvent flow rate of 0.25 mL/min, as previously described [40]. The phenolic compounds were identified based on their UV–vis and mass spectra and retention times and comparison with authentic standards and previous reports [6,41] (Supplementary Table S1). Quantification of the phenolic compounds was based on calibration curves of external standards and expressed as mg per 100 mL. Anthocyanins were quantified as equivalents of cyanidin-3-galactoside at 520 nm, flavonols as rutin at 360 nm, flavan-3-ols as (+)-catechin at 280 nm and HCAs as chlorogenic acid at 320 nm. Chromatograms at the different wavelengths are given in Supplementary Fig. S1.

### 2.5 Colour analysis

The colour of the juices was measured using a digital colour measurement system (DigiEye, VeriVide Ltd., Leicester, UK). The juice (1 mL) in white plastic cups was placed in a lightbox with standardized daylight (D65) and diffuse lighting and photographed with a calibrated digital camera (Nikon D7000, 35 mm lens, Nikon Corp., Tokyo, Japan). Colour measurements of the pictures were performed with DigiPix software (version 2.63). The colour was based on CIE (Commission International de l’Eclairage), and the colour components L*a*b. L* is a measure of lightness; lower values indicate darker colour (0 = black), and higher values indicate lighter colour (100 = white). The hue angle (Equation (1)) designates colour shade, where low values (°Hue = 0°) indicate a red-bluish colour and high values (°Hue = 90°) indicate a yellow colour. Chroma (Equation (2)) is a measure of colour saturation, where high values indicate pure colours. The absolute colour difference of the samples after heat treatment and storage was calculated as ΔE (Equation (3)).

(1) °Hue=arctan(b*/a*)

(2) $$Chroma={\left(a{*}^{2}+b{*}^{2}\right)}^{1/2}$$

(3) $$\Delta E={\left({\left(L_{0}*-L*\right)}^{2}\;+\;{\left(a_{0}*-a*\right)}^{2}\;+\;{\left(b_{0}-b*\right)}^{2}\right)}^{1/2}$$

where L_0_*, a_0_* and b_0_* are the values at time 0.

### 2.6 Kinetic calculations

The degradation of phenolic compounds was assumed to follow first-order reaction kinetics, and the reaction rate constant (*k*) and the half-lives (t_1/2_) were determined by Equations (4), (5)).

(4) $$\ln\left(C_{t}/C_{0}\right)=-kt$$

(5) $$t_{1/2}=\ln\left(2\right)/k$$

where C_t_ is the concentration at time t and C_0_ is the initial concentration. *k* was determined as the slope of the graph ln(C_t_/C_0_) against heating time (hours) or storage time (weeks).

### 2.7 Statistical analyses

The differences in chemical composition in the newly made juices (US, SUK, ACE and SUC) were determined by one-way analysis of variance (ANOVA), followed by Tukey pairwise comparisons test with significant level *p* < 0.05. The effects of juice (Juice; with no or different sweeteners), temperature (Temp), and their interaction (Juice x Temp) on half-lives of phenolic compounds were determined by ANOVA, a general linear model (GLM). In the thermal experiment, the factors were Juice (fixed, 2 levels; US and SUK) and Temp (fixed, 3 levels; 75, 85 and 95 °C). Due to no or very small changes in phenolic compounds at 50 °C in the experimental period (2 h), the results at 50 °C were not included in the ANOVA analysis. In the storage experiment, the factors were Juice (fixed, 4 levels; US, SUK, ACE and SUC) and Temp (fixed, 2 levels; 6 and 22 °C). Significant differences (*p* < 0.05) between average responses of Juice x Temp were determined by Tukey pairwise comparisons test. The statistical analysis was performed using Minitab® (version 19.2020.1).

## 3 Results and discussion

### 3.1 Characterization of the experimental juices

#### 3.1.1 Soluble solids, pH, and titratable acidity

The soluble solids (SS), pH and titratable acidity (TA) in the unsweetened juice (US) were 9.3° Brix, 2.68 and 1.38 g/100 g, respectively (Table 1). The values were comparable with previous findings in lingonberries [42,43]. The juice with added sucrose (SUK) had a higher SS, which reflected the 10% added sucrose. TA in SUK juice was approximately 90% of the value in US, which is in accordance with the dilution of this juice.

**Table 1:** Soluble solids (SS), pH, titratable acidity (TA) and concentrations (mg/100 mL) of phenolic compounds in newly made unsweetened juice (US), juice with 10% sucrose (SUK), juice with 0.017% sucralose (SUC) and juice with 0.057% acesulfame K (ACE)^a^,^b^.

	US	SUK	ACE	SUC
SS (° Brix)	9.3 ± 0.0 b	18.2 ± 0.0 a	9.3 ± 0.0 b	8.9 ± 0.0 c
pH	2.68 ± 0.01	2.67 ± 0.02	2.68 ± 0.02	2.68 ± 0.01
TA (g/100 g)	1.38 ± 0.00 a	1.22 ± 0.00 c	1.38 ± 0.01 a	1.32 ± 0.00 b
Anthocyanins				
Cy-gal	41.1 ± 0.9 ab	38.6 ± 1.2 b	41.6 ± 0.8 ab	43.4 ± 2.2 a
Cy-glu	2.29 ± 0.02 ab	2.15 ± 0.02 b	2.29 ± 0.02 ab	2.35 ± 0.11 a
Cy-ara	6.61 ± 0.05 ab	6.22 ± 0.08 b	6.59 ± 0.07 ab	6.81 ± 0.33 a
Flavonols				
Q-gal	4.20 ± 0.09 ab	3.94 ± 0.14 b	4.20 ± 0.09 ab	4.41 ± 0.19 a
Q-ara	2.06 ± 0.30	1.98 ± 0.33	2.34 ± 0.16	2.52 ± 0.22
Q-rham	5.39 ± 0.07 ab	5.05 ± 0.08 b	5.32 ± 0.05 ab	5.48 ± 0.21 a
Q-(HMG)-rham	3.81 ± 0.06 ab	3.58 ± 0.05 b	3.75 ± 0.03 ab	3.88 ± 0.16 a
Q	3.70 ± 0.53	3.35 ± 0.45	3.12 ± 0.18	3.13 ± 0.29
Flavan-3-ols				
Cat	25.8 ± 0.6 ab	24.0 ± 0.7 b	26.1 ± 1.3 ab	26.7 ± 1.5a
Epicat	9.7 ± 1.0	9.0 ± 1.4	10.4 ± 0.1	10.1 ± 0.9
A-type dimer	21.5 ± 1.3	20.4 ± 1.1	20.3 ± 1.7	22.5 ± 1.0
B-type dimer	22.2 ± 0.1 a	20.9 ± 0.3 b	22.2 ± 0.2 a	22.5 ± 0.6 a
HCAs				
p-CA-hex	3.27 ± 0.04 ab	3.03 ± 0.13 b	3.28 ± 0.03 ab	3.34 ± 0.06 a
3-CQA	1.48 ± 0.02 ab	1.40 ± 0.03 b	1.47 ± 0.01 ab	1.49 ± 0.03 a
FA-hex 1	2.45 ± 0.02 a	2.31 ± 0.02 b	2.43 ± 0.02 a	2.48 ± 0.06 a
FA-hex 2	2.44 ± 0.04	2.24 ± 0.21	2.46 ± 0.02	2.50 ± 0.05
p-CA	1.50 ± 0.07	1.39 ± 0.04	1.43 ± 0.01	1.45 ± 0.01

a Abbreviations: cy, cyanidin; gal, galactoside; glu, glucoside; ara, arabinoside; Q, quercetin; rham, rhamnoside; HMG, 3-hydroxy-3-methylglutaroyl.

b The concentrations are mean values and standard deviations of triplicate samples (n = 3). Values in a row with different letters are different (*p* < 0.05) based on Tukey pairwise comparisons test.

#### 3.1.2 Phenolic compounds

The three most abundant anthocyanins in the juices were cyanidin-3-galactoside (79%), cyanidin-3-arabinoside (13%) and cyanidin-3-glucoside (4%), with a total concentration of ca. 50 mg/100 mL in the newly made unsweetened juice (Table 1). The quantitative and qualitative contents of anthocyanins were in line with previous results in lingonberries [15] and lingonberry juice [41]. The predominant flavonols were quercetin-3-rhamnoside (28%), -galactoside (22%), -(3-hydroxy-3-methylglutaroyl) (HMG)-rhamnoside (20%) and -arabinoside (11%), and the quercetin aglycone (19%), with a total concentration of ca. 19 mg/100 mL (Table 1). Assessment of flavan-3-ols using the HPLC-DAD-MS method was difficult due to coelution with other phenolic compounds and low molar absorptivity. (+)-Catechin, (−)-epicatechin, a B-type dimer and an A-type dimer were quantified and used to determine the response of flavan-3-ols to the treatments. The major HCAs in the juices were a *p*-coumaric acid hexoside, chlorogenic acid, two ferulic acid hexosides and *p*-coumaric acid, with a total concentration of approximately 11 mg/100 mL. Overall, the phenolic composition in the juices was in accordance with previous reports [41,44]. The concentrations in the SUK juice were 91–95% of the concentrations in the unsweetened juice (US) and the juices sweetened with the high-intensity sweeteners (ACE and SUC), which was in accordance with the dilution effect (ca. 93% of the initial values are expected).

#### 3.1.3 Colour

**Fig. 1:**
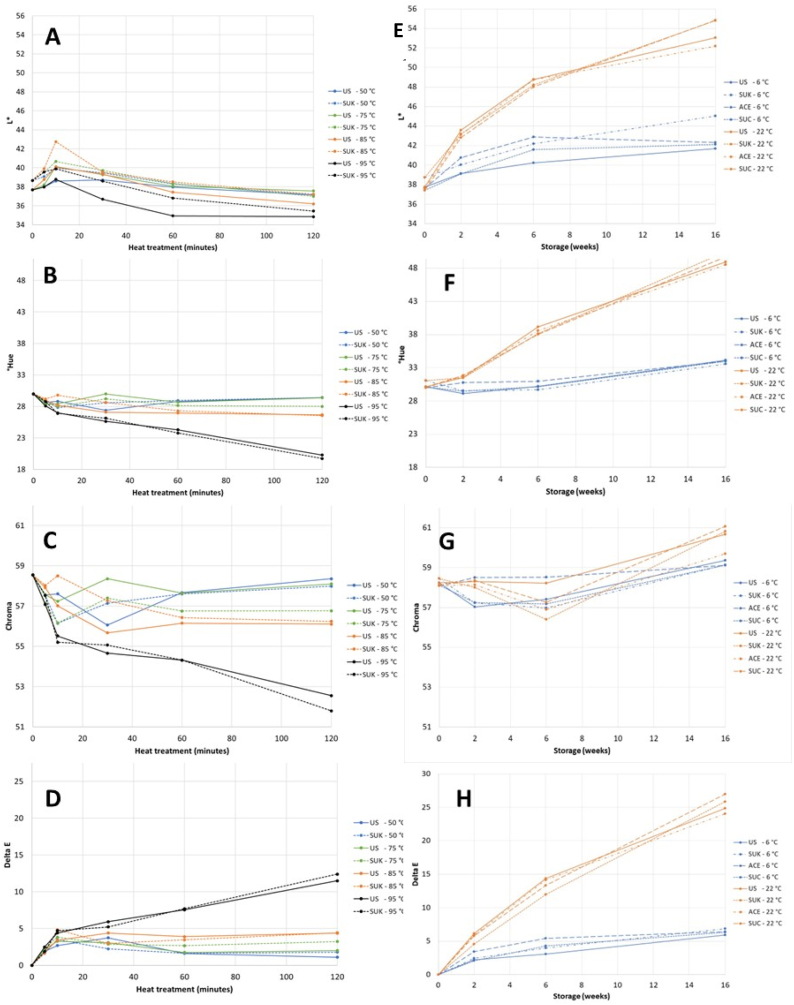
The colour parameters, L*, °Hue, Chroma and ΔE, in unsweetened juice (US) and juice with 10% sucrose (SUK) during thermal treatment at 50, 75, 85 and 95 °C for 120 min (A–D) and in US, SUK, juice with 0.017% sucralose (SUC) and juice with 0.057% acesulfame K (ACE) during storage at 6 and 22 °C for 16 weeks (E–H). (For interpretation of the references to colour in this figure legend, the reader is referred to the Web version of this article.)

The lingonberry juice had a dark, red colour. In the newly made unsweetened juice (US), the L*-value was 37.7. The juice sweetened with sucrose (SUK) was slightly lighter (L* = 38.7) than the other juices (Fig. 1). The addition of sweetener did not affect °Hue or Chroma.

### 3.2 The stability of phenolic compounds

#### 3.2.1 The stability of anthocyanins during thermal treatment

**Fig. 2:**
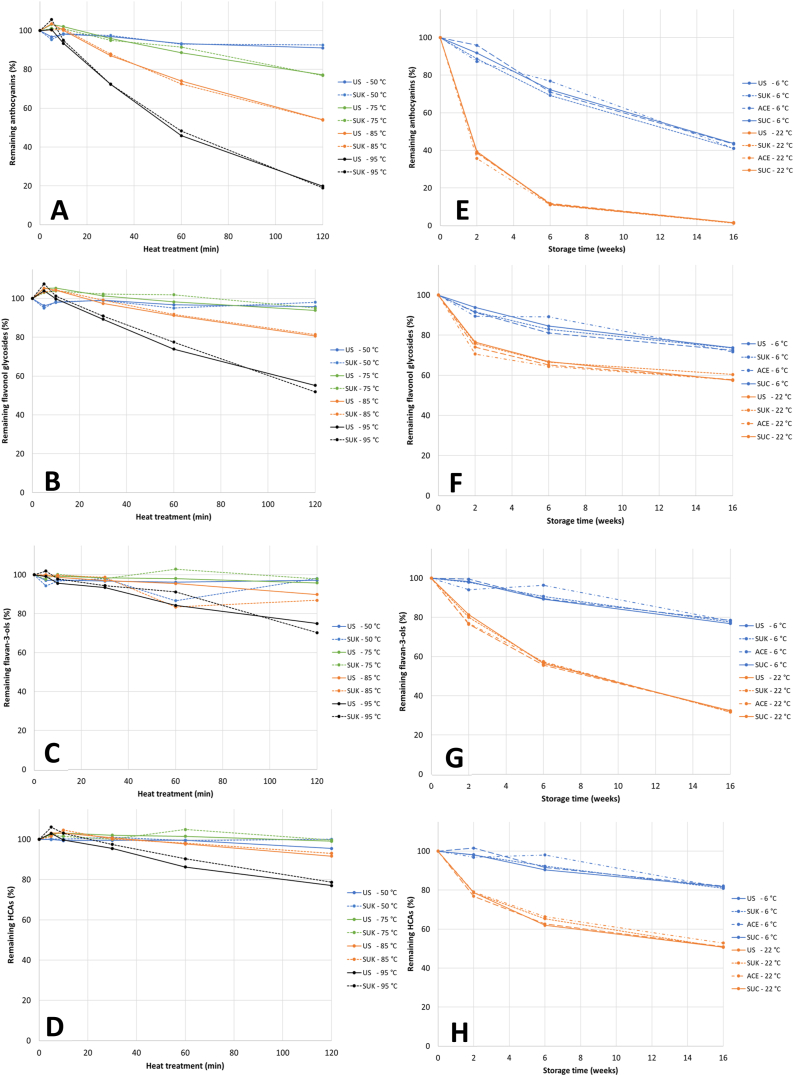
Percent remaining anthocyanins (A), flavonol glycosides (B), flavan-3-ols (C) and HCAs (D) in unsweetened juice (US) and juice with 10% sucrose (SUK) during thermal treatment at 50, 75, 85 and 95 °C for 120 min. Percent remaining anthocyanins (E), flavonol glycosides (F), flavan-3-ols (G) and HCAs (H) in US, SUK, juice with 0.017% sucralose (SUC) and juice with 0.057% acesulfame K (ACE) during storage at 6 and 22 °C for 16 weeks. Anthocyanins includes all anthocyanins. Flavonol glycosides includes four quercetin glycosides. Flavan-3-ols are the sum of (+)-catechin, (−)-epicatechin, a B-type dimer and a A-type dimer. The HCAs are the sum of a *p*-coumaric acid hexoside, chlorogenic acid and two ferulic acid hexosides.

The first 10 min of heat treatment, irrespective of temperature, had no effect on the total concentration of anthocyanins (Fig. 2A). Anthocyanin concentrations in the juices heated at 85 and 95 °C declined rapidly after 10 min. Anthocyanins in the juices heated at 75 °C were stable for 60 min, while anthocyanins in the juices heated at 50 °C were quite stable throughout the experimental period. This is accordance with other studies on degradation of anthocyanins in berry juices, demonstrating high stability of anthocyanins at 50–60 °C [45,46]. After 120 min at 50 °C, ca. 90% of the initial anthocyanins remained, whereas in juices heated at 75 °C, 85 °C and 95 °C for 120 min, ca. 77%, 55% and 20% of the initial anthocyanins remained, respectively (Fig. 2A). The degradation of the anthocyanins followed first-order reaction kinetics, and the half-lives of total anthocyanins in the juices heated at 75, 85 and 95 °C were on average 4.7, 2.0 and 0.8 h, respectively (Table 2). Two mechanisms are suggested to be responsible for thermal degradation of anthocyanins 1) hydrolysis of the 3-glycoside linkage to form the unstable aglycon and 2) nucleophilic attack of water on the flavylium cation to form a carbinol followed by the formation of a chalcone through opening of the C ring [23]. The stability of anthocyanins was significantly affected by temperature but not addition of sucrose. Sucrose was shown to stabilize anthocyanins in a model system due to the ability to bind water and thereby hindering nucleophilic attack of water on the flavylium cation [47]. The protective effect increased with sucrose concentration from about 20% sucrose. The sucrose concentration in the present study may thus be too low to exert any stabilizing effect by water binding. Similarly, no significant effects of supplementation of sucrose (50 g/L) to juices of elderberry, strawberry and black carrots on half-lives of anthocyanins were seen upon heating at 95 °C [48]. In other studies, diverging results are reported on the effect of sucrose on anthocyanin stability. In cornelian cherry juice, sucrose (12%) stabilized anthocyanins when heated at 75 °C [30] and in blackberry juice a small protective effect was seen with addition of 10% sucrose at 50 °C, while at 70 and 90 °C sucrose slightly destabilized anthocyanins [33]. Addition of sucrose (10–20%) decreased thermostability (75–95 °C) of anthocyanins in extracts of blackcurrants [29]. The results suggests that the influence of sucrose on thermal stability of anthocyanins is small and dependent of food matrix and the temperature applied. Lingonberries have low pH and low concentrations of ascorbic acid [43], which should be favourable for the stability of anthocyanins [24]. Despite this, anthocyanins in lingonberry juice were less stable than anthocyanins in many other products, as the half-lives of anthocyanins in juices or extracts of diverse berries varied from 1 to 15 h at 70–80 °C [23]. One of the reasons for this finding could be the nature of the anthocyanins in lingonberries. Lingonberries contain simple anthocyanins with only monosaccharides bound to the aglycon, and anthocyanins with more complex sugars and cinnamic acids attached are shown to be more stable than monoglycosidic anthocyanins [16,18,29,48]. Among the simple anthocyanins, pentosides were less stable than hexosides [21,22,48], and glucosides were more stable than galactosides in cranberries [21]. In accordance with this, we found that cyanidin-3-arabinoside was less stable than cyanidin-3-galactoside and cyanidin-3-glucoside, and glucoside was more stable than galactoside during heat treatment (Table 2). After 120 min at 95 °C, 7% of the initial cyanidin-3-arabinoside was present in the juice, while 18 and 32% of cyanidin-3-galactoside and cyanidin-3-glucoside remained, respectively (Supplementary Table S2). After extensive heating, small amounts of the aglycon cyanidin as well as its degradation products protocatechuic acid and phloroglucinaldehyde were detected in the juices (results not shown).

**Table 2:** Half-lives (t_1/2_) of phenolic compounds in unsweetened juice (US) and juice with 10% sucrose (SUK) heated at 75, 85, and 95 °C for 2 h^a^,^b^.

	t_1/2_ (hours) at 75 °C		t_1/2_ (hours) at 85 °C		t_1/2_ (hours) at 95 °C	
	US	SUK	US	SUK	US	SUK
Total anthocyanins	4.6 a	4.8 a	2.0 b	2.0 b	0.8 c	0.8 c
Cy-gal	4.8 a	5.0 a	2.1 b	2.1 b	0.8 c	0.8 c
Cy-glu	6.1 a	6.4 a	2.9 b	2.8 b	1.2 c	1.1 c
Cy-ara	3.4 a	3.5 a	1.4 b	1.4 b	0.5 c	0.5 c
Total flavonol glycosides	na^c^	na	5.4 a	5.7 a	2.2 b	2.2 b
Q-gal	na	na	9.9 a	10.4 a	3.4 b	3.4 b
Q-ara	3.9 a	5.2 a	1.4 b	1.5 b	0.6 b	0.7 b
Q-rham	na	na	4.9 a	5.2 a	1.5 b	1.6 b
Q-(HMG)-rham	na	na	na	na	6.9 a	6.8 a
Total flavan-3-ols	na	na	na	na	4.4 a	3.3 a
Cat	na	na	na	na	7.7 a	5.5 a
Epicat	na	na	na	na	3.3 a	3.0 a
A-type dimer	na	na	3.4 a	2.9 a	1.7 b	1.6 b
B-type dimer	na	na	na	na	na	na
Total HCAs (not p-CA)	na	na	na	na	4.9 a	5.1 a
p-CA-hex	na	na	4.2 a	4.7 a	1.4 b	1.5 b
3-CQA	na	na	na	na	9.4 a	8.0 a
FA-hex 1	na	na	na	na	na	na
FA-hex 2	na	na	na	na	na	na

a Abbreviations: cy, cyanidin; gal, galactoside; glu, glucoside; ara, arabinoside; Q, quercetin; rham, rhamnoside; HMG, 3-hydroxy-3-methylglutaroyl.

b Values in a row with different letters are different (*p* < 0.05) based on Tukey pairwise comparisons test.

c na = not applicable due to no or small changes during the thermal treatment for 2 h.

#### 3.2.2 The stability of flavonols, flavan-3-ols and hydroxycinnamic acids during thermal treatment

The flavonol glycosides were stable during heating at 50 and 75 °C for 2 h (Fig. 2B). The stability of flavonols was significantly affected by temperature but not the addition of sucrose (Table 2). The half-lives of total flavonol glycosides at 85 and 95 °C were on average 5.6 and 2.2 h, respectively. Quercetin-3-arabinoside and quercetin-3-rhamnoside were the least stable flavonol glycosides, with half-lives of 0.7 and 1.6 h at 95 °C, respectively, while quercetin-3-(HMG)-rhamnoside was the most stable flavonol glycoside. Half-life of quercetin-3-glucose previously reported in freeze-dried sour cherry paste (49 min at 80–90 °C) [49] was somewhat lower than found in the present study. The influence of the sugar moiety on the degradation of quercetin glycosides was the same as observed during thermal treatment (180 °C) of dried flavonol solutions, where especially the 3-rhamnoside degraded rapidly [50]. As the quercetin glycosides degraded, the concentration of quercetin, the aglycon, increased. Similarly, the concentration of quercetin steadily increased during thermal treatment in the present study. At 85 °C the concentration of quercetin was doubled after 2 h and at 95 °C the concentration was more than doubled after 1 h (Supplementary Table S2). This finding is also in accordance with a previous study demonstrating that a combination of heat and low pH resulted in significant formation of flavonol aglycones in cranberries [21].

The flavan-3-ols were quite stable during thermal treatment (Fig. 2C), and half-lives at 50–85 °C could not be calculated, except for the A-type dimer, which was the most unstable of the flavan-3-ols quantified (Table 2). The half-lives of this compound were on average 3.1 and 1.6 h at 85 and 95 °C, respectively. Addition of sucrose did not influence the stability of the flavan-3-ols.

There was no effect of sucrose addition on the stability of HCAs in the lingonberry juice during thermal treatment (Fig. 2D, Table 2). A *p*-coumaric acid hexoside was the least stable HCA, with a half-life of 1.4 h at 95 °C. Chlorogenic acid had half-life of 8–9 h at 95 °C, which was considerable longer than the half-life of about 1 h found for chlorogenic acid in freeze-dried sour cherry paste at 80–90 °C [49]. The two ferulic acid hexosides were stable at all temperatures. The concentration of *p*-coumaric acid increased during thermal treatment and was doubled after 2 h at 95 °C (Supplementary Table S2). The increase in *p*-coumaric acid is explained by hydrolysis of *p*-coumaric glycosides, such as the *p*-coumaric acid hexoside, decreasing at approximately the same rate as *p*-coumaric acid increased.

The stability of flavonols, flavan-3-ols and hydroxycinnamic acids in berry products, especially during thermal treatment are, in opposition to for anthocyanins, hardly studied. One reason for this can be the greater importance of anthocyanins, contributing both to colour and health promoting properties of the products. Another reason can be that the anthocyanins are the least stable among the phenolic compounds [[20], [21], [22],^49^]. In accordance with previous findings, the anthocyanins were the least stable of the phenolic compounds also in the present study (Table 2, Table 3). Anthocyanins, in opposition to the other phenolic compounds, undergo reversible structural transformations as a function of pH, and at low pH and increasing temperatures the equilibrium is shifted to the more unstable chalcone form [23].

**Table 3:** Half-lives (t_1/2_) of phenolic compounds in unsweetened juice (US), juice with 10% sucrose (SUK), juice with 0.017% sucralose (SUC) and juice with 0.057% acesulfame K (ACE) stored at 6 and 22 °C for 16 weeks^a^,^b^.

	t_1/2_ (weeks) at 6 °C				t_1/2_ (weeks) at 22 °C			
	US	SUK	ACE	SUC	US	SUK	ACE	SUC
Total anthocyanins	13.2 a	12.5 a	12.6 a	12.7 a	2.8 b	2.6 b	2.7 b	2.7 b
Cy-gal	11.5 a	10.7 a	10.9 a	11.1 a	2.3 b	2.2 b	2.3 b	2.3 b
Cy-glu	29.0 a	28.7 a	28.4 a	27.0 a	4.2 b	4.0 b	4.1 b	4.2 b
Cy-ara	27.0 a	26.6 a	26.6 a	25.2 a	3.7 b	3.5 b	3.5 b	3.7 b
Total flavonol glycosides	37.0 a	38.4 a	36.6 a	33.6 ab	23.4 c	26.4 bc	25.7 c	21.7 c
Q-gal	12.1 a	11.3 a	10.5 a	11.8 a	6.0 b	7.6 b	7.9 b	6.7 b
Q-ara	11.0 a	11.3 a	10.6 a	10.0 a	9.6 a	10.3 a	9.2 a	8.0 a
Q-rham	na	na	na	na	78.0 a	90.8 a	85.6 a	76.3 a
Q-(HMG)-rham	na^c^	na	na	na	60.9 a	70.9 a	65.9 a	58.5 a
Total flavan-3-ols	41.4 a	42.2 a	47.8 a	42.0 a	10.1 b	9.9 b	10.1 b	10.0 b
Cat	36.2 a	39.9 a	36.9 a	35.3 a	9.5 b	9.9 b	9.3 b	9.4 b
Epicat	36.2 a	42.3 a	32.6 a	35.7 a	13.3 b	14.3 b	12.8 b	11.0 b
A-type dimer	35.5 a	31.3 a	40.5 a	38.6 a	7.2 b	6.5 b	7.8 b	7.9 b
B-type dimer	72.8 a	73.6 a	74.4 a	68.5 a	12.6 b	12.6 b	12.3 b	12.7 b
Total HCAs (not p-CA)	54.5 a	52.7 a	55.3 a	51.2 a	18.1 b	18.2 b	19.4 b	17.5 b
p-CA-hex	29.0 a	27.8 a	30.6 a	27.8 a	7.8 b	6.9 b	8.4 b	7.7 b
3-CQA	55.7 a	50.7 a	63.6 a	53.4 a	20.7 b	20.3 b	23.5 b	21.3 b
FA-hex 1	81.7 a	91.1 a	98.0 a	80.3 a	22.2 b	29.6 b	26.0 b	23.8 b
FA-hex 2	na	na	na	na	50.4 a	49.0 a	47.0 a	40.9 a

a Abbreviations: cy, cyanidin; gal, galactoside; glu, glucoside; ara, arabinoside; Q, quercetin; rham, rhamnoside; HMG, 3-hydroxy-3-methylglutaroyl.

b Values in a row with different letters are different (*p* < 0.05) based on Tukey pairwise comparisons test.

c na = not applicable due to no or very small changes during the storage period (16 weeks).

#### 3.2.3 The stability of anthocyanins during storage

The stability of anthocyanins during storage was significantly affected by temperature but not the addition of sweeteners (Table 3). The lack of effect of sweeteners on anthocyanin stability may not be surprising as the concentration of sucrose and the other sweeteners were quite low (see discussion in 3.2.1). In addition are diverging results on the effect of sweetener on anthocyanin stability during storage previously reported. Sucrose (12%) and acesulfame K (0.1%) destabilized anthocyanins in cornelian cherry juice, and more at 25 °C than at 2 °C [30] and sucrose (20%) destabilized anthocyanins isolated from sour cherries [51], while in blackberry juice and sour cherry puree the addition of sucrose (7–10%) slightly increased stability of anthocyanins during storage at 4 °C [34,52].

The half-lives of total anthocyanins in the juices were on average 12.8 and 2.7 weeks at 6 and 22 °C, respectively (Table 3). The values were comparable with those in mixtures of black currant juices, which had half-lives of total anthocyanins of 8.2–19.4 weeks at 4 °C and 2.0–2.8 weeks at 20 °C [13], and in the lower range of values reported for various berry juices, which had half-lives of 12.3–32.5 weeks and 2.2–6.7 weeks at 4 °C and 21 °C, respectively [14]. The half-lives of total anthocyanins found in the present study was also lower than half-lives of total anthocyanins found in most of the lingonberry jams added sucrose and other sweeteners [32] and in blackberry jams with different sugars and sugar alcohols [31]. The higher stability of anthocyanins in jams compared with juices may be due to stabilizing effects of a more complex and viscous matrix with lower water mobility. After two weeks at 22 °C, 37–40% of the initial anthocyanins remained, which was comparable to the remaining content after 16 weeks at 6 °C (Fig. 2E). After 16 weeks at 22 °C, less than 2% of the initial anthocyanins remained in the juices. Previously, 13% retention of anthocyanins in lingonberry juice after 14.7 weeks at ambient temperature was reported [28]. Cyanidin-3-galactoside was the least stable anthocyanin during storage with half-lives of, on average, 11.1 and 2.3 weeks at 6 and 22 °C, respectively, compared with cyanidin-3-glucoside and cyanidin-3-arabinoside, with half-lives of 25–29 weeks at 6 °C and ca. 4 weeks at 22 °C (Table 3). The reason for the more severe breakdown of cyanidin-3-galactoside during storage is probably side-activities of the enzyme formulation used to reduce mash viscosity during preparation of the juice, as the processing enzymes were not inactivated by pasteurized before heat treatment or storage of the juice. Some commercial pectinase mixtures used for juice processing are shown to contain impurities of glucosidases, arabinosidases and galactosidases, with the latter being by far the most detrimental for anthocyanins [17,53,54]. After enzymatic treatment of bilberries with enzymatic preparations, the anthocyanin profile of bilberries was altered, and hardly any anthocyanidin galactosides remained in the juices [17]. During thermal treatment at high temperatures, it is expected that enzymes are inactivated, while at 50 °C enzymes are active, and accordingly, cyanidin-3-galactoside was the least stable anthocyanin at this temperature (Supplementary Table S2). Additionally, a decrease in cyanidin-3-galactoside was found after enzymatic mash treatment of lingonberries at 50 °C [41]. This strengthens the hypothesis that galactoside side-activities of the pectinase formulation used in the production of the juice was the cause of the severe degradation of cyanidin-3-galactoside during storage. With inactivation of galactosidase, the stability of cyanidin-3-galactoside would be expected to be like that of cyanidin-3-glucoside; thus, as cyanidin-3-galactoside is the major anthocyanin (ca. 80%) in lingonberries, the half-lives of total anthocyanins in lingonberry juice might be doubled with inactivation of galactosidase.

#### 3.2.4 The stability of flavonols, flavan-3-ols and hydroxycinnamic acids during storage

In accordance with previous studies [18,[20], [21], [22],^31^], the flavonols were more stable than anthocyanins also during storage (Fig. 1 and Table 3). The concentrations of flavonol glycosides had decreased 10–30% after two weeks of storage and continued to steadily decrease in the storage period (Fig. 2F). Worth noting, the difference in stability when stored cold compared with at room temperature was lower for flavonol glycosides than for the other phenolic compounds analysed (Fig. 2E–H). The stability of flavonol glycosides was significantly affected by temperature but not by the addition of sweeteners (Table 3). The half-lives of total flavonol glycosides were on average 36 and 24 weeks when stored at 6 and 22 °C, respectively. In blackcurrant juice, there was no change in the concentration of flavonol glycosides after one year at 4 °C [20]. In chokeberry juice, most flavonols were very stable, and there was more than 90% retention after 6 months at 25 °C [22]. In both of those studies, the juices were stored in glass bottles, which have lower oxygen permeability than the polypropylene tubes used in the present study, which may provide higher stability against oxidation. The different stability of flavonols in lingonberry juice compared with juices of blackcurrants and chokeberries might also be caused by internal factors, such as the structure of the flavonols, other ingredients in the juice, and degrading enzymes. There were huge differences in the stability of the various quercetin glycosides during storage. Quercetin-3-rhamnoside and quercetin-3-(HMG)-rhamnoside were stable during storage, while quercetin-3-galactoside and quercetin-3-arabinoside had half-lives of in average 11 weeks at 6 °C and 8 weeks at 22 °C (Table 3). It is plausible that galactoside side-activities of the pectinase formulation used in the production of the juice is responsible for the degradation of quercetin-3-galactoside, as it is for cyanidin-3-galactoside. During storage, the concentration of quercetin in the juices first increased and reached about 6 mg/100 g fw after 2 weeks at 22 °C and 6 weeks at 6 °C, then declined (Supplementary Table S3). After 16 weeks at 22 °C, only approximately 0.1 mg/100 mL of quercetin was present in the juices. Quercetin was previously shown to degrade into several products, including protocatechuic acid [55], which was also found in the present study (results not shown).

After 16 weeks at 6 °C and 22 °C, about 80% and 30% of the flavan-3-ols remained in the juices, respectively (Fig. 2G). Temperature but not the addition of sweeteners affected the stability of the flavan-3-ols (Table 3). The half-lives of the sum of the four flavan-3-ols were 43 and 10 weeks when stored at 6 °C and 22 °C, respectively. The A-type dimer was the least stable of the flavan-3-ols during storage at 22 °C, with half-life of 7.3 weeks.

The addition of sweeteners did not affect the stability of the HCAs during storage (Fig. 2H, Table 3). The concentration of free *p*-coumaric acid increased during storage and the concentration was doubled after two weeks at 22 °C (Supplementary Table S3). Released free hydroxycinnamic acids was also detected in black currant juice during storage [20]. The half-lives of total HCAs, not including *p*-coumaric acid, were approximately one year when stored at 6 °C and 18.4 weeks when stored at 22 °C (Table 3). The stability of HCAs in other berry juices were higher than those in our study [20,22], which could be caused by the factors previously mentioned.

### 3.3 The stability of colour

There were minor changes in colour during heat treatment, except for the juices heated at 95 °C, and not affected by the addition of sweeteners (Fig. 1A–D). The juices heated at 95 °C for 60 and 120 min were darker (lower L*), more bluish (lower °Hue) and had lower colour purity (Chroma) than the newly made juices. The changes in Chroma and °Hue were more pronounced than changes in L*. The °Hue results are consistent with those of a study of purified anthocyanins, where lower hue angles were found after heating at 95 °C for up to 7 h [48]. The overall colour differences (ΔE) in that study, with values from 5.5 to 8.9 after 60 min at 95 °C, were also in accordance with our findings (Fig. 1D). However, in contrast to our study, the lightness and chroma increased during heat treatment in that study. The reason for these contrasting results could be that purified anthocyanins were studied in that prior study; thus, there were no other compounds that could interact with anthocyanins to influence colour development.

#### 3.3.1 The stability of colour during storage

During storage, in contrast to during thermal treatment, L*, °Hue and Chroma increased; that is, the juices became lighter, more yellowish and had more colour saturation (Fig. 1E–G). In accordance with our findings, in a prior study, °Hue and L* increased in lingonberry juice after 14.7 weeks at ambient temperature [28]; however, Chroma decreased. The changes in colour were most pronounced in the juices stored at 22 °C for six weeks or longer, with an overall colour difference ΔE > 10 (Fig. 1H). The colour of the juices during storage was not affected by the addition of sweeteners.

#### 3.3.2 Anthocyanins and colour development

Anthocyanins are responsible for the red colour of lingonberries, but there was no correlation between anthocyanin content and juice colour when the results after heat treatment and storage were combined. Although there was a negative (logarithmic) correlation between total anthocyanin content and absolute colour change (ΔE) both after thermal treatment and storage (results not shown), the colour development after thermal treatment and storage was different. The reason for the different colours after thermal treatment and storage is probably that oxidation and polymerization products formed during storage give a brownish colour to the stored products, while these compounds are not produced in the short duration of the thermal treatment. The bluish and darker colour occurring in the juices at the harshest thermal treatments might be caused by pyranoanthocyanins formed by the reaction between anthocyanins and free hydroxycinnamic acids, such as *p*-coumaric acid, liberated during heating. In accordance with this, the formation of pyranoanthocyanins from hydroxycinnamic acids and anthocyanins has been demonstrated in juices of strawberries and raspberries [56], and the addition of hydroxycinnamic acids has been shown to decrease the °Hue and L*-values of several berry juices, including lingonberry juice [28].

## 4 Conclusion

The addition of sucrose (10%), sucralose (0.017%) or acesulfame K (0.057%) did not influence the stability of phenolic compounds or the colour of lingonberry juice during heat treatment and storage and can thus be added to lingonberry juice probably without negatively affecting the stability of health-related and sensory quality of the juice. This result, however, cannot exclude the possibility that the addition of higher concentrations of sweeteners or the addition of other sweeteners might influence the stability of phenolic compounds or the colour of lingonberry juice. Heat treatment typically employed in juice processing had no effect on the quality of lingonberry juice. However, heating at higher temperatures (85–95 °C) for more than 10 min should be avoided. The colour of lingonberry juice was affected differently by thermal treatment and storage. After thermal treatment, the juices were darker and bluer with a lower chromaticity, while after storage, the juices became lighter, more yellow and had higher chromaticity. Anthocyanins were the least stable of the phenolic compounds quantified. One of the reasons for the extensive losses of anthocyanins during storage is probably the galactosidase side-activities of the pectinase formulation used in juice production, leading to selective degradation of the major anthocyanin in lingonberries, cyanidin-3-galactoside. Processing enzymes should be inactivated to avoid enzymatic hydrolysis of the anthocyanins and other phenolic compounds during storage. This study shows the importance of storage at low temperature to preserve phenolic compounds and the colour of berry juices.

## Author contribution statement

Kjersti Aaby: Conceived and designed the experiments; Performed the experiments; Analyzed and interpreted the data; Wrote the paper.

Mathias Rudolf Amundsen: Performed the experiments; Analyzed and interpreted the data; Wrote the paper.

## Data availability statement

Data will be made available on request.

## Funding sources

This work was supported by the 10.13039/501100005416Research Council of Norway (grant no. 294797) and The Norwegian Fund for Research Fees for Agricultural Products (grants no. 314599 and 314318).

## Declaration of competing interest

The authors declare that they have no competing financial interests or personal relationships that could have appeared to influence the work reported in this paper.

## References

[1] Paassilta M.M., Moisio S., Jaakola L., Häggman H. (2009).

[2] Kowalska K. (2021). Lingonberry (*Vaccinium vitis-idaea* L.) Fruit as a source of bioactive compounds with health-promoting effects- a review. Int. J. Mol. Sci..

[3] Del Bo C., Bernardi S., Marino M., Porrini M., Tucci M., Guglielmetti S., Cherubini A., Carrieri B., Kirkup B., Kroon P., Zamora-Ros R., Liberona N.H., Andres-Lacueva C., Riso P. (2019). Systematic review on polyphenol intake and health outcomes: is there sufficient evidence to define a health-promoting polyphenol-rich dietary pattern?. Nutrition.

[4] Grosso G., Godos J., Currenti W., Micek A., Falzone L., Libra M., Giampieri F., Forbes-Hernandez T.Y., Quiles J.L., Battino M., La Vignera S., Galvano F. (2022). The effect of dietary polyphenols on vascular health and hypertension: current evidence and mechanisms of action. Nutrition.

[5] Hellström J., Törrönen A.R., Mattila P.H. (2009). Proanthocyanidins in common food products of plant origin. J. Agric. Food Chem..

[6] Bujor O.C., Ginies C., Popa V.I., Dufour C. (2018). Phenolic compounds and antioxidant activity of lingonberry (*Vaccinium vitis-idaea* L.) leaf, stem and fruit at different harvest periods. Food Chem..

[7] Ek S., Kartimo H., Mattila S., Tolonen A. (2006). Characterization of phenolic compounds from lingonberry (*Vaccinium vitis-idaea*). J. Agric. Food Chem..

[8] Cassidy A. (2018). Berry anthocyanin intake and cardiovascular health. Mol. Aspect. Med..

[9] Rodriguez-Mateos A., Heiss C., Borges G., Crozier A. (2014). Berry (poly)phenols and cardiovascular health. J. Agric. Food Chem..

[10] Todaro A., Cavallaro R., La Malfa S., Continella A., Gentile A., Fischer U.A., Carle R., Spagna G. (2016). Anthocyanin profile and antioxidant activity of freshly squeezed pomegranate *(Punica Granatum* L.) juices of Sicilian and Spanish provenances. Ital. J. Food Sci..

[11] Giuffre A.M., Zappia C., Capocasale M. (2017). Physicochemical stability of blood orange juice during frozen storage. Int. J. Food Prop..

[12] Aaby K., Mazur S., Nes A., Skrede G. (2012). Phenolic compounds in strawberry (*Fragaria* x *ananassa* Duch.) fruits: composition in 27 cultivars and changes during ripening. Food Chem..

[13] Dobson G., McDougall G.J., Stewart D., Cubero M.A., Karjalainen R.O. (2017). Effects of juice matrix and pasteurization on stability of black currant anthocyanins during storage. J. Food Sci..

[14] Hellström J., Mattila P., Karjalainen R. (2013). Stability of anthocyanins in berry juices stored at different temperatures. J. Food Compos. Anal..

[15] Lee J., Finn C.E. (2012). Lingonberry (*Vaccinium vitis-idaea* L.) grown in the Pacific Northwest of North America: anthocyanin and free amino acid composition. J. Funct.Foods.

[16] Sadilova E., Stintzing F.C., Kammerer D.R., Carle R. (2009). Matrix dependent impact of sugar and ascorbic acid addition on color and anthocyanin stability of black carrot, elderberry and strawberry single strength and from concentrate juices upon thermal treatment. Food Res. Int..

[17] Buchert J., Koponen J.M., Suutarinen M., Mustranta A., Lille M., Torronen R., Poutanen K. (2005). Effect of enzyme-aided pressing on anthocyanin yield and profiles in bilberry and blackcurrant juices. J. Sci. Food Agric..

[18] Neves C.M.B., Pinto A., Goncalves F., Wessel D.F. (2021). Changes in elderberry (*Sambucus nigra* L.) juice concentrate polyphenols during storage. Appl. Sci.-Basel.

[19] McLellan M.R., Padilla-Zakour O.I., Barrett D.M., Somogyi L., Ramaswamy H.S. (2005). Processing Fruits.

[20] Mäkila L., Laaksonen O., Alanne A.L., Kortesniemi M., Kallio H., Yang B.R. (2016). Stability of hydroxycinnamic acid derivatives, flavonol glycosides, and anthocyanins in black currant juice. J. Agric. Food Chem..

[21] White B.L., Howard L.R., Prior R.L. (2011). Impact of different stages of juice processing on the anthocyanin, flavonol, and procyanidin contents of cranberries. J. Agric. Food Chem..

[22] Wilkes K., Howard L.R., Brownmiller C., Prior R.L. (2014). Changes in chokeberry (*Aronia melanocarpa* L.) polyphenols during juice processing and storage. J. Agric. Food Chem..

[23] Oancea S. (2021). A review of the current knowledge of thermal stability of anthocyanins and approaches to their stabilization to heat. Antioxidants.

[24] Cavalcanti R.N., Santos D.T., Meireles M.A.A. (2011). Non-thermal stabilization mechanisms of anthocyanins in model and food systems-An overview. Food Res. Int..

[25] Skrede G., Wrolstad R.E., Lea P., Enersen G. (1992). Color stability of strawberry and blackcurrant syrups. J. Food Sci..

[26] Howard L.R., Prior R.L., Liyanage R., Lay J.O. (2012). Processing and storage effect on berry polyphenols: challenges and Implications for bioactive properties. J. Agric. Food Chem..

[27] Cai D.B., Li X.S., Chen J.L., Jiang X.W., Ma X.Q., Sun J.X., Tian L.M., Vidyarthi S.K., Xu J.W., Pan Z.L., Bai W.B. (2022). A comprehensive review on innovative and advanced stabilization approaches of anthocyanin by modifying structure and controlling environmental factors. Food Chem..

[28] Rein M.J., Heinonen M. (2004). Stability and enhancement of berry juice color. J. Agric. Food Chem..

[29] Rubinskiene M., Viskelis P., Jasutiene I., Viskeliene R., Bobinas C. (2005). Impact of various factors on the composition and stability of black currant anthocyanins. Food Res. Int..

[30] Moldovan B., David L. (2020). Influence of different sweeteners on the stability of anthocyanins from cornelian cherry juice. Foods.

[31] Benedek C., Bodor Z., Merrill V.T., Kókai Z., Gere A., Kovacs Z., Dalmadi I., Abrankó L. (2020). Effect of sweeteners and storage on compositional and sensory properties of blackberry jams. Eur. Food Res. Technol..

[32] Scrob T., Varodi S.M., Vintila G.A., Casoni D., Cimpoiu C. (2022). Estimation of degradation kinetics of bioactive compounds in several lingonberry jams as affected by different sweeteners and storage conditions. Food Chem. X.

[33] Kopjar M., Pilizota V. (2011). Prevention of thermal degradation of anthocyanins in blackberry juice with addition of different sugars. Cyta -J. Food.

[34] Kopjar M., Jaksic K., Pilizota V. (2012). Influence of sugars and chlorogenic acid addition on anthocyanin content, antioxidant activity and color of blackberry juice during storage. J. Food Process. Preserv..

[35] Martinsen B.K., Aaby K., Skrede G. (2020). Effect of temperature on stability of anthocyanins, ascorbic acid and color in strawberry and raspberry jams. Food Chem..

[36] Hubbermann E.M., Heins A., Stockmann H., Schwarz K. (2006). Influence of acids, salt, sugars and hydrocolloids on the colour stability of anthocyanin rich black currant and elderberry concentrates. Eur. Food Res. Technol..

[37] Laaksonen O., Knaapila A., Niva T., Deegan K.C., Sandell M. (2016). Sensory properties and consumer characteristics contributing to liking of berries. Food Qual. Prefer..

[38] Rocha I.F.D., Bolini H.M.A. (2015). Different sweeteners in passion fruit juice: ideal and equivalent sweetness. LWT-Food Sci. Technol..

[39] Yebra-Biurrun M.C., Worshold P., Townshend A., Poole C. (2005). Encyclopedia of Analytical Science.

[40] Aaby K., Grimmer S., Holtung L. (2013). Extraction of phenolic compounds from bilberry (*Vaccinium myrtillus* L) press residue: effects on phenolic composition and cell proliferation. Lwt-Food Sci. Technol..

[41] Marsol-Vall A., Kelanne N., Nuutinen A., Yang B., Laaksonen O. (2021). Influence of enzymatic treatment on the chemical composition of lingonberry (*Vaccinium vitis-idaea*) juice. Food Chem..

[42] Viljakainen S., Visti A., Laakso S. (2002). Concentrations of organic acids and soluble sugars in juices from Nordic berries. Acta Agric. Scand. Sec. B-Soil Plant Sci..

[43] Skrede G., Martinsen B.K., Wold A.B., Birkeland S.E., Aaby K. (2012). Variation in quality parameters between and within 14 Nordic tree fruit and berry species. Acta Agr. Scand. B-S P.

[44] Kelanne N., Laaksonen O., Seppala T., Yang W., Tuukkanen K., Loponen J., Yang B.R. (2019). Impact of cyclodextrin treatment on composition and sensory properties of lingonberry (*Vaccinium vitis-idaea*) juice. Lwt-Food Sci. Technol..

[45] Wang W.D., Xu S.Y. (2007). Degradation kinetics of anthocyanins in blackberry juice and concentrate. J. Food Eng..

[46] Kechinski C.P., Guimaraes P.V.R., Norena C.P.Z., Tessaro I.C., Marczak L.D.F. (2010). Degradation kinetics of anthocyanin in blueberry juice during thermal treatment. J. Food Sci..

[47] Tsai P.J., Hsieh Y.Y., Huang T.C. (2004). Effect of sugar on anthocyanin degradation and water mobility in a roselle anthocyanin model system using O-17 NMR. J. Agric. Food Chem..

[48] Sadilova E., Stintzing F.C., Carle R. (2006). Thermal degradation of acylated and nonacylated anthocyanins. J. Food Sci..

[49] Zoric Z., Dragovic-Uzelac V., Pedisic S., Kurtanzek Z., Garofulic I.E. (2014). Kinetics of the degradation of anthocyanins, phenolic acids and flavonols during heat treatments of freeze-dried sour cherry marasca paste. Food Technol. Biotechnol..

[50] Rohn S., Buchner N., Driemel G., Rauser M., Kroh L.W. (2007). Thermal degradation of onion quercetin glucosides under roasting conditions. J. Agric. Food Chem..

[51] Turkyilmaz M., Hamzaoglu F., Ozkan M. (2019). Effects of sucrose and copigment sources on the major anthocyanins isolated from sour cherries. Food Chem..

[52] Nowicka P., Wojdylo A. (2016). Stability of phenolic compounds, antioxidant activity and colour through natural sweeteners addition during storage of sour cherry puree. Food Chem..

[53] Wightman J.D., Wrolstad R.E. (1995). Anthocyanin analysis as a measure of glycosidase activity in enzymes for juice processing. J. Food Sci..

[54] Wightman J.D., Wrolstad R.E. (1996). β-glucosidase activity in juice-processing enzymes based on anthocyanin analysis. J. Food Sci..

[55] Buchner N., Krumbein A., Rohn S., Kroh L.W. (2006). Effect of thermal processing on the flavonols rutin and quercetin. Rapid Commun. Mass Spectrom..

[56] Rein M.J., Ollilainen V., Vahermo M., Yli-Kauhaluoma J., Heinonen M. (2005). Identification of novel pyranoanthocyanins in berry juices. Eur. Food Res. Technol..

